# New antithrombotic drugs: a revolution in stroke management

**Published:** 2012-03

**Authors:** Alan Bryer

**Affiliations:** Division of Neurology and Stroke Unit, Groote Schuur Hospital and University of Cape Town, South Africa

Embolism of cardiac origin accounts for 20% of ischaemic strokes. Atrial fibrillation is by far the most common cause of cardioembolic stroke, and anticoagulation is the treatment generally indicated for secondary, and in many cases, primary prevention.^1^ The decision to prescribe warfarin is usually based on an accurate assessment of the likely absolute annual risk of stroke without warfarin, and whether or not such benefits of warfarin treatment are likely to outweigh the risk of bleeding associated with its use.

For more than 20 years, the use of warfarin has been the cornerstone of antithrombotic therapy for patients with TIA or ischaemic stroke due to cardioembolism, particularly those associated with atrial fibrillation. Warfarin remains the commonest anticoagulant used worldwide (although other similar vitamin K antagonists are prescribed in many countries).

Adjusted-dose warfarin anticoagulation with an international normalised ratio (INR) range between 2.0 and 3.0 is significantly more effective than antiplatelet therapy for preventing recurrent stroke in patients with atrial fibrillation and results in a risk reduction of between 60 and 68% compared to placebo.^2^,^3^ By contrast, the most commonly used alternative to warfarin is aspirin, which provides substantially less-consistent benefit and reduces the risk of recurrent stroke and other major vascular events in patients with atrial fibrillation by only 17 to 21%.^4^,^5^

Similarly, combination antiplatelet therapy with aspirin and clopidogrel is not as effective as warfarin and is associated with a significant increase in major bleeding.^6^ Furthermore, although current data indicate that combination treatment with aspirin and clopidogrel does result in a greater reduction in major vascular events when compared with aspirin alone, this is offset by an increase in major haemorrhages. The absolute benefit of oral anticoagulation with warfarin versus antiplatelet therapy increases as patients with atrial fibrillation get older because stroke risk increases with age while the relative efficacy of oral anticoagulation therapy to prevent ischaemic stroke does not change.^7^

Despite the efficacy and affordability of warfarin, many patients with cardioembolic stroke or TIA are not treated with this agent because it is perceived to be inconvenient or hazardous. Although the benefits of oral anticoagulation with warfarin are supported by a high degree of evidence for stroke prevention due to cardioembolic stroke, there are many disadvantages associated with its use. The long-term efficacy and safety of warfarin depends on maintaining a narrow range of anticoagulation intensity (INR 2.0–3.0) and this may be compromised by the patient’s dietary intake, exposure to other drugs, and co-existing illnesses. Consequently, many drug-compliant patients are not well controlled and require regular monitoring of the INR.

The need for sustained patient monitoring is not only inconvenient for the patient but also requires adequate healthcare infrastructure, which is often lacking in developing countries. For instance, patients who have residual disability after a cardio-embolic stroke may experience significant difficulties in attending clinics where their INR can be monitored and their warfarin dose adjusted accordingly. This problem is often compounded in rural areas where the distances patients have to travel to clinics may be considerable and infrastructure at such clinics for INR monitoring may be lacking.

As patients on warfarin need to be within the target INR range in order to achieve benefit, there is also an increased risk for serious bleeding complications when the target INR is exceeded. In a *post-hoc* analysis of the RE-LY trial, a wide variation in the time in therapeutic range (TTR) across participating countries persisted despite efforts to improve the generally poor quality of INR control seen in many trials. This ranged from a high 77% in Sweden to as low as between 41 and 58% in 16 other countries, mostly Asia, Eastern Europe, South America and South Africa.^8^

An audit of anticoagulation was undertaken in a cohort of patients attending a prothrombin clinic at a tertiary South African hospital in order to determine the TTR on dose-adjusted warfarin. Patients were included in the audit if the indication for warfarin was atrial fibrillation or a mechanical valve replacement and they had been on warfarin for at least one month. Of the 190 patients included in the analyses, the mean TTR was 55.5%, with a complication rate of 8.4% (5.8% bleeding, 2.6% thrombotic). The TTRs for the majority of the patients in this study were lower than acceptable, at the lower end of published norms and associated with a high complication rate. Neither clinic attendance nor time on warfarin correlated with the TTR. The results of this audit indicate that the level of anticoagulation was inadequate in the majority of patients treated with warfarin at this large clinic.^9^ It is likely that these results reflect the situation in many clinics in the developing world.

Numerous drug and dietary interactions compound the problem of warfarin’s narrow therapeutic range and the difficulties in achieving adequate TTR. Warfarin can interact with a multitude of commonly prescribed drugs (such as statins, various antibiotics, non-steroidal anti-inflammatory agents and some of the most popular over-the-counter analgesics such as paracetamol and aspirin). Given the problems associated with its use, clinicians are frequently compelled to prescribe less efficacious antiplatlet agents for prevention of cardioembolic stroke.

The advent of the direct thrombin inhibitors and factor Xa inhibitors represents a quantum leap forward in the long-term prevention of recurrent stroke of cardiac origin. The two overwhelming advantages of the new agents are that they exhibit stable pharmacokinetics, obviating the need for coagulation monitoring or dose titration, and that they lack clinically significant food or drug interactions. Additional advantages are that they offer fixed once- or twice-daily oral dosing and a rapid onset of action. It seems likely that, in time, these agents will replace warfarin as treatment of choice for the prevention of cardioembolic stroke.

September 2009 heralded the publication of the first of three important studies in which the front runners of these new agents, dabigatran, and subsequently apixaban and rivaroxaban, were each compared to warfarin in patients with atrial fibrillation, in order to determine whether or not these new agents provided more consistent and predictable anticoagulation than warfarin for a primary endpoint of stroke or systemic embolism. Results from these trials indicate that all three novel anticoagulants are either non-inferior or superior to warfarin in reducing the risk of stroke and systemic embolisation.^10^-^12^ Furthermore, all three drugs have either an equivalent or reduced risk of major bleeding and intracranial haemorrhage compared with warfarin. However, there is continued debate and discussion in the literature concerning the variability in the trial designs of these studies, particularly pertaining to issues such as the differences in the case mix affecting stroke risk (e.g. differences in the CHADS_2_ scores, prevalence of prior stroke, patient age, whether or not patients were warfarin naive, and the interpretation of the TTR data).

Although the current trials show favourable safety profiles for these newer agents, long-term data are still required, as most patients with atrial fibrillation require lifelong oral anticoagulation. Nevertheless, these agents appear to provide a number of significant benefits over warfarin, and potential patients should be informed of these in order to make informed choices. On the other hand, there are a number of concerns which will need to be addressed.

Widespread use of these newer agents is expected in the future once they are approved by the relevant regulatory authorities. Inevitably, the potential risk for overdose will increase in this population, particularly among the elderly, and there is currently no easy way of detecting this with routine coagulation tests. There is also currently no solid evidence to guide the management of bleeding complications that can occur with these newer agents. The thrombin time and ecarin clotting time do illustrate a linear response to serum dabigatran concentration, but are not readily available. Consequently, many patients already taking and tolerating warfarin, with good INR control, may reasonably prefer not to switch to dabigatran or one of the factor Xa inhibitors until there is more clarity on these issues.

A major limiting factor for the future widespread use of the newer anticoagulants in the developing world will be their high cost compared to warfarin. In evaluating the health economics of introducing these newer therapies into the public health domain of African countries, the cost of these drugs will need to be compared not only with the cost of warfarin but also with the cost and availability of INR-monitoring facilities. Furthermore, the cost of non-compliance and inadequate TTR on warfarin treatment, as well as associated complications of warfarin therapy will need to be carefully considered. The analysis of cost-effectiveness of the new drugs will need to include these ramifications for stroke prevention so that their true risk–benefit can be properly assessed.

Fortunately there are a number of competing drugs in this new class, with other similar products in development (betrixaban, edoxaban). This is likely to drive down the prices of these new agents, allowing for more widespread use. These drugs also have the potential to expand the number of patients eligible for oral anticoagulant therapy, including those patients with atrial fibrillation who are unable or unwilling to use warfarin.

Dabigatran has already received regulatory approval in the United States for use in patients with atrial fibrillation and it has rapidly entered clinical practice. It is likely that apixaban and rivaroxaban will also get regulatory approval and the debate in the literature concerning their comparative efficacy and safety will continue.

Many physicians are reluctant to prescribe warfarin for elderly patients in atrial fibrillation for various reasons (e.g. concerns for risk of falls, history of previous bleeding) despite clear evidence of increased benefit in these patients compared with younger patients. These physicians would likely have fewer reservations about prescribing one of the newer agents. The consistent anticoagulant effect achieved with the new oral anticoagulants may also translate into greater efficacy and safety due to avoidance of the frequent sub- and supra-therapeutic drug levels, which are common with warfarin and the other vitamin K antagonists.
